# Placental Dysfunction As a Key Element in The Pathogenesis of Preeclampsia

**DOI:** 10.34763/devperiodmed.20172104.309316

**Published:** 2018-01-02

**Authors:** Henning Schneider

**Affiliations:** 1 Department of Obstetrics and Gynaecology, Inselspital, Bern University Hospital, Bern, Switzerland; 2 Institute of Biochemistry and Molecular Medicine, University of Bern, Bern, Switzerland

**Keywords:** preeclampsia, early placental development - vascular protection, early placental development - vascular dysfunction, syncytiotrophoblastic stress, antioxidant defence, predisposition to endothelial disease

## Abstract

Placental pathology is associated with major pregnancy disorders and the concept of the Great Placental Syndromes encompasses disorders of placentation, such as preeclampsia with and without fetal growth restriction, preterm labor, preterm premature rupture of membranes, late spontaneous abortion, and placental abruption. Preeclampsia is divided between the early and late onset variety and placental dysfunction is a central feature in the pathogenesis of both. In the early onset type, syncytiotrophoblastic stress seems to be related to an inherent defect of the trophoblast. Vascular protection of early placental development is replaced by vascular dysfunction. In late onset preeclampsia, maternal factors, such as genotypic predisposition to endothelial disease, and an impairment of antioxidant defence with a limited capacity of the maternal clearing system to cope with the increasing charge of apoptotic cell debris, are at the center of pathogenesis. Syncytiotrophoblastic stress in late pregnancy has been related to molecular senescence and late onset preeclampsia may be viewed as an exaggeration of normal placental ageing.

## Introduction

Placental pathology is associated with major pregnancy disorders. The concept of the Great Obstetrical Syndromes encompasses disorders of placentation, such as preeclampsia with and without fetal growth restriction, preterm labor, preterm premature rupture of membranes, late spontaneous abortion, and placental abruption [1].

Preeclampsia is a serious pregnancy-specific disorder, affecting 10 million women annually worldwide. It is associated with high maternal and fetal morbidity and mortality. The clinical picture is characterized by arterial hypertension and proteinuria. It is a systemic disease affecting different organs in the mother, leading over time to renal insufficiency, hepatic dysfunction, thrombocytopenia and pulmonary edema, among others. Involvement of the central nervous system is reflected by cerebral edema, hyperreflexia and/or visual impairment [2].

Approximately 15% of the cases of prematurity and 25% of intrauterine growth restriction are linked to preeclampsia. Apart from unfavourable consequences on pregnancy outcome, preeclampsia-related morbidity may be associated with important negative effects on the long-term health of the mother and the child, including increased risks for cardiovascular and metabolic diseases [3].

The July 1973 issue of “Medical World News” featured “The great eclampsia mystery or the case of the empty plaque” as the title story and referred to the famous plaque on the wall of the Chicago “Lying In Hospital” with the names of four “obstetrical pioneers” [4]. The still empty center field is reserved for “eclampsia’s eradicator” and the question was raised whether ”toxemia of pregnancy” – as preeclampsia/eclampsia was called for many years in the United States – would disappear before anyone finds its cause and cure.

In recent years we have learnt a lot about the disease, but “toxemia of pregnancy” has not disappeared and we still know neither its “cause nor cure”. However, the central role of placental dysfunction in the pathogenesis of both early and late onset preeclampsia has generally been accepted nowadays.

## Early onset preeclampsia

Early onset preeclampsia develops in 2 stages: soon after implantation signs of deficient placentation appear. At a later stage this is followed by a clinical picture known as the maternal syndrome based on systemic vascular inflammation [5]. The early onset variety appears before 34 weeks and accounts for between 5% to 20% of all cases. It is characterised by reduced placental volume, abnormal uterine and umbilical artery Doppler findings, and signs of fetal growth restriction. Systemic vascular inflammation leads to the dysfunction of many organs in the mother and, consequently, adverse maternal and neonatal outcomes are common.

## Normal and abnormal development of the uteroplacental circulation

**Fig. 1:**
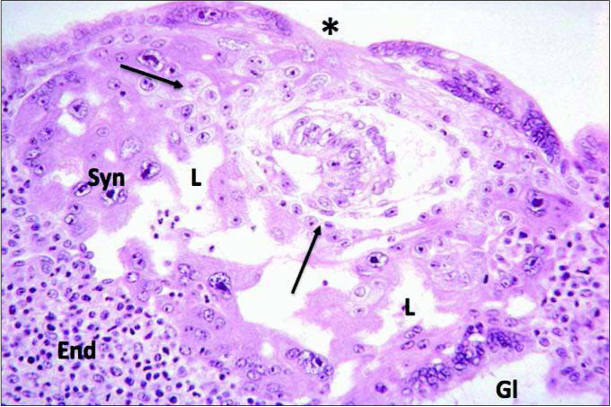
This picture is from the Carnegie Collection and shows normal development of a human pregnancy at Day 9 post conception. Implantation is almost complete and the conceptus is at the blastocyst stage showing two cell lineages, the embryoblast and the trophoblast. By fusion of uninucleate cytotrophoblast the multinucleate syncytiotrophoblast (Syn) is formed. Lacunae (L) inside the syncytiotrophoblast are forerunners of the intervillous space, which at this stage has no open connection with the uteroplacental vasculature. At the right lower corner the syncytiotrophoblast has eroded the wall of an endometrial gland (Gl), which releases its secretions into the lacuna. Adapted from [6].

In normal human pregnancy, on Day 9 after conception the process of implantation is almost complete and the conceptus is at the blastocyst stage showing two cell lineages, the embryoblast and the trophoblast. By fusion of uninucleate cytotrophoblast cells the multinucleate syncytiotrophoblast (STB) is formed. Lacunae inside the STB are forerunners of the intervillous space, which at this early stage demonstrates no open connection with the uteroplacental vasculature (Figure 1) [6]. Interestingly, at the right lower corner of the Figure, the STB has eroded the wall of an endometrial gland, which can thus release its secretions directly into the lacuna.

On Day 12 after conception (corresponding to Week 4 after the last menstrual period) the cytotrophoblast penetrates into the STB forming primitive villi. Upon contact of these villi with the maternal decidua the trophoblast proliferates and eventually differentiates into a villous and an extravillous variant. Before maternal blood flow inside the intervillous space is triggered, low tissue oxygen activates hypoxia inducible factor (HIF) -1α thus stimulating the production of transforming growth factor TGFβ3. Towards the end of the first trimester, i.e. with the start of circulation of maternal blood inside the intervillous space, PO_2_ rises, HIF-1α is down-regulated, and TGFβ3 activity becomes suppressed. The extravillous trophoblast switches from a proliferative to a more invasive phenotype [7].

Remodelling of the spiral arteries with the transformation of the distal segment from a narrow high resistance vessel in the non-pregnant endometrium to the dilated low resistance spiral artery of pregnancy is an important feature of normal placentation. Following early implantation, the spiral arteries remain plugged by invading cytotrophoblast. Unplugging starts at about 8 weeks of gestation when chorionic villi have matured enough to sustain oxidative stress resulting from direct exposure to oxygenated maternal blood [8].

**Fig. 2:**
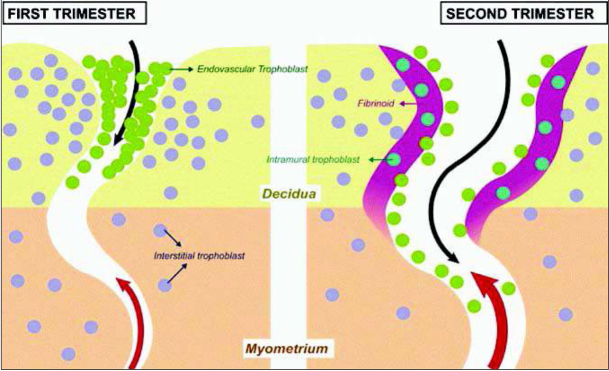
Conversion of the spiral arteries. On the left: a spiral artery in the first trimester, endovascular trophoblast is shown in green and extravascular or interstitial trophoblast in blue. On the right: a spiral artery in the second trimester with complete transformation. Both, endovascular trophoblast acting from the lumen of the vessel and the interstitial trophoblast acting from the outside are involved in the process of transformation. Adapted from [9].

On the left of Figure 2, a spiral artery is shown in an early stage of transformation with endovascular trophoblast in green and extravascular or interstitial trophoblast in blue [9]. In the second trimester shown on the right the transformation is already complete. Deep endovascular invasion of the spiral arteries by the trophoblast is associated with a full remodelling of the walls of the arteries, including their deep myometrial segments. Both endovascular trophoblast acting from the lumen of the vessel and the interstitial trophoblast acting from the outside are involved in the process of transformation. The latter together with maternal leucocytes interact with the extracellular matrix of the vascular wall in the spiral arteries in replacing smooth muscle cells. Uterine natural killer (NK) cells and various enzymes of the matrix metalloproteinase family are also involved in the process of remodelling [10].

**Fig. 3:**
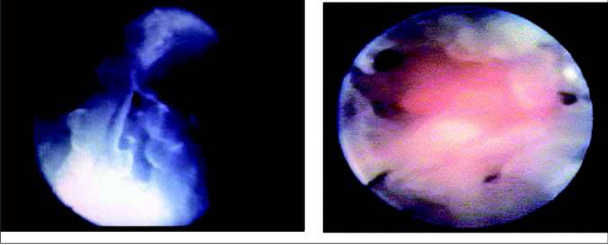
Start of maternal blood flow in hysteroscopy video recordings. The picture on the left is taken before 10 weeks when the intervillous space contains clear fluid but no blood. The picture on the right is taken at the end of the first trimester, after the spiral arteries have been opened and maternal blood has entered the intervillous space. Adapted from [11].

Using hysteroscopy Schaaps and Hustin were the first to demonstrate that in normal pregnancy maternal blood appears inside the intervillous space only towards the end of the first trimester (Figure 3) [11]. The picture on the left is from a hysteroscopy done before 10 weeks. The intervillous space contains clear fluid but no blood. The picture on the right is from a hysteroscopy done at the end of the first trimester after the spiral arteries have opened and maternal blood is circulating in the intervillous space. Many common complications of pregnancy are associated with a deficient physiological conversion of the mother’s spiral arteries supplying the placenta [1].

**Fig. 4:**
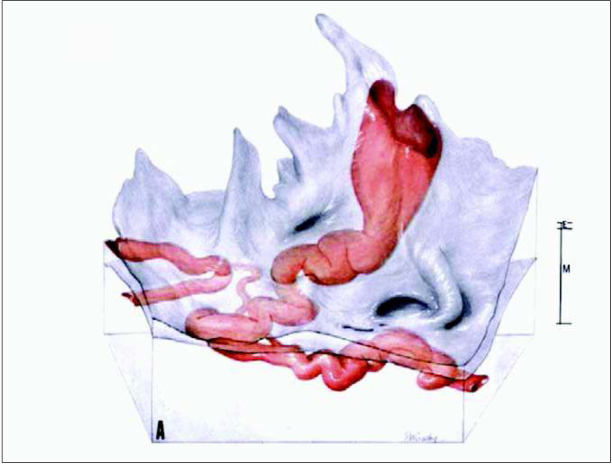
Three-dimensional reconstruction based on serial sections of a specimen from a hysterectomy performed after delivery at term. Myometrial (M) and endometrial (E) segments of the spiral artery are shown. Before opening into the intervillous space the terminal coil of the vessel is funnel-shaped and has a diameter of 2-3 mm. Adapted from [12].

**Fig. 5:**
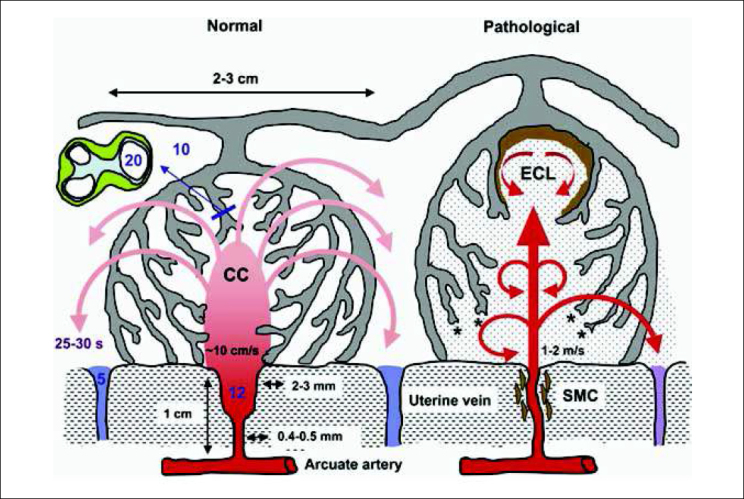
Based on this reconstruction Graham Burton and colleagues speculated on rheological consequences of the conversion of the spiral arteries. On the left, conditions in normal pregnancy are shown. The funnel-shaped terminal dilation of the spiral artery serves to slow down the speed of uteroplacental blood flow to 10 cm/s as it enters the intervillous space. From the central cavity of the lobule the maternal blood evenly disperses through the villous tree. An estimated transit time of 25-30 s is sufficient to allow diffusion of an adequate amount of oxygen from erythrocytes to the trophoblast. On the right, conditions found in abnormal pregnancies are shown. With incomplete conversion of the spiral arteries, maternal blood enters the intervillous space at a much higher speed of 1 to 2 m/s, anchoring villi are torn off the basal plate and echogenic cysts form underneath the chorionic plate. The oxygen exchange is impaired by the reduced transit time. Shedding of microparticles from the surface of the villous trophoblast into the intervillous space is also increased. Adapted from [12].

The impact of this transformation on different segments of a spiral artery passing through the myometrium and the endometrium before opening into the intervillous space is presented in Figure 4. This three-dimensional reconstruction is based on serial sections of a specimen from a hysterectomy performed after delivery at term and was taken from Harris and Ramsey. The terminal coil of the vessel is funnel-shaped and has a diameter of 2-3 mm, which is at least 4 times wider than the myometrial or endometrial segments (0.4-0.5 mm) [12]. Based on this reconstruction, the group of Burton speculated on rheological consequences of the conversion of the spiral arteries [12]. In normal pregnancy, the funnel-shaped terminal dilation of the spiral artery slows down the uteroplacental blood flow entering the intervillous space from 1 to 2 m/s to 10 cm/s as shown on the left of Figure 5. Therefore, maternal blood evenly disperses from the central cavity of the lobule through the villous tree. The estimated transit time of the maternal blood flow before reaching the venous exit of the intervillous space was 25-30 s, which is sufficient to allow the diffusion of an adequate amount of oxygen from maternal erythrocytes to the trophoblast.

With defective remodelling, the constant low-pressure flow of uteroplacental perfusion changes to a more pulsatile high-pressure flow at the increased speed of 1 to 2 m/s. Anchoring villi are torn off the basal plate and echogenic cysts form underneath the chorionic plate, as shown on the right of Figure 5. The disturbed flow of maternal blood causes oxidative stress of the villous trophoblast with increased secretion of various factors, including the antiangiogenic soluble *vascular endothelial growth factor* receptor fms-like tyrosine kinase 1 (also termed sFlt-1 or sVEGFR-1) that together induce the maternal syndrome [5].

**Fig. 6:**
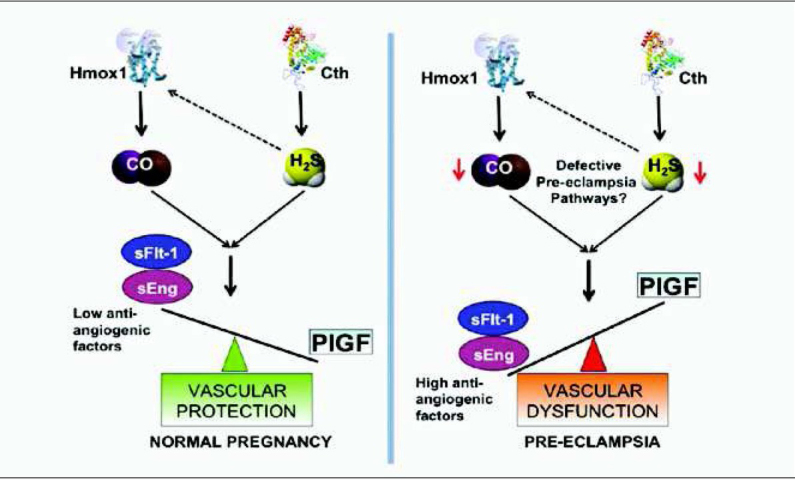
In normal pregnancy, early placental development is characterized by the proliferation of villi, a process which is driven by angiogenesis. The trophoblastic enzymes hem oxygenase 1 (Hmox1) and cystathionine γ-lyase (Cth) generate gaseous signalling molecules carbon monoxide (CO) and hydrogen sulphide (H_2_S), which together with placental growth factor (PlGF), another product of trophoblast, provide a strong proangiogenic effect, also known as “Vascular Protection”. Ahmed and Ramma have proposed that in early onset preeclampsia intrinsic alterations in the biology of the trophoblast lead to an impairment of “Vascular Protection”. Due to an increased secretion of fms like tyrosine kinase 1 as soluble VEGF receptor (sFlt-1) as well as soluble endoglin together with a reduced production of PlGF “Vascular Protection” of normal pregnancy is replaced by “Vascular Dysfunction” of preeclampsia. Adapted from [13].

Ahmed and Ramma have proposed that angiogenesis acts as driving force for the proliferation of placental villi in early placentation of normal pregnancy [13]. The trophoblastic enzymes hem oxygenase 1 (Hmox1) and cystathionine γ-lyase (Cth) generate gaseous signalling molecules carbon monoxide (CO) and hydrogen sulphide (H_2_S) which, together with placental growth factor (PlGF) (another important product of the trophoblast), have a strong proangiogenic effect. This has been described as “Vascular Protection” and is shown on the left of Figure 6. In early onset preeclampsia, intrinsic alterations in the biology of the trophoblast lead to an impairment of “Vascular Protection”. Namely, due to increased secretion of sFlt-1, as well as soluble endoglin, together with a reduced production of (PlGF), “Vascular Protection” of normal pregnancy becomes “Vascular Dysfunction” of preeclampsia, shown on the right of Figure 6.

Deficient placentation with early unplugging together with restricted invasion of spiral arteries by extravillous trophoblast lead to impaired remodelling and predispose to early onset preeclampsia, which is commonly associated with intrauterine growth restriction (IUGR) of the fetus or IUGR without preeclampsia. The clinical syndrome of early onset preeclampsia is caused by shedding of dysfunctional membrane fragments of STB into the maternal blood circulating inside the intervillous space leading to systemic endothelial activation, which, together with inflammatory response, results in leukocyte and complement activation and disturbed coagulation [14]. Placental oxidative stress and endoplasmic reticulum stress may accelerate cellular senescence of trophoblast, which has been described in placentas associated with early, but not late, onset preeclampsia [15, 16]. In line, magnetic resonance imaging has demonstrated a reduced perfusion of the placenta in cases of early onset, but not late onset, disease [17]. A change in the phosphodiester/phosphomonoester ratio has been described as a sign of this accelerated ageing as studied by ^31^P-*magnetic resonance* spectroscopy [18].

The uterine immune system plays an important role in regulating the phenomenon of placentation. Deficient invasion of the extravillous trophoblast into the decidua is a common denominator of the Great Obstetrical Syndromes (preeclampsia, growth restriction, stillbirth, and preterm labor) [1]. The decidua basalis is densely populated by uterine Natural Killer cells (uNK), a unique type of lymphocytes. Killer Immunoglobulin-like receptors (KIR) expressed on the surface of these cells allow them to recognize and respond to paternally-derived HLA-C molecules of the extravillous trophoblast. Particular combinations of maternal KIR and fetal (paternal) HLA-C genotypes characterize each pregnancy and certain combinations have a strong inhibitory effect on the invasion of spiral arteries by extravillous trophoblast and are associated with an increased risk for preeclampsia [19].

## Late onset preeclampsia

Late onset preeclampsia appears after 34 weeks and accounts for at least 80% of all cases [3]. It is predominantly seen in conditions with increased placental tissue mass, such as diabetes or multiple pregnancy, or with increased placental surface, which is related to hypoxic conditions, such as maternal anemia or living at high altitude. Such situations are associated with an increased release of syncytial knots [20]. Uterine and umbilical artery Doppler examinations tend to be normal, as is fetal weight. The effect on maternal and neonatal outcome is less severe compared to the early onset disease.

An increase in placental mass together with the release of syncytial knots are observed in apparently normal pregnancies and have previously been considered markers of placental maturity. However, since syncytial knots can be induced in placental explants by hypoxia or oxidative stress, Redman and Staff suggested that they should be considered markers of STB stress. Further, they pointed out that signs of STB stress are commonly found in late pregnancies, an observation suggesting that the duration of pregnancy is restricted by placental capacity. Parallels between postmaturity and late onset preeclampsia strengthen the concept of considerable overlap in pathogenesis [5]. The STB stress developing in late pregnancy has been related to molecular senescence as a result of progressive mismatch between maternal perfusion and fetal demands [21]. Therefore, late onset preeclampsia may be viewed as an exaggeration of normal placental ageing [15].

For both early and late onset preeclampsia, the STB stress is a central feature of pathogenesis. A major difference is that, in the early onset type, the STB stress seems to be related to an inherent defect of the trophoblast leading to an impairment of early placentation. In the late onset type, limitations of placental capacity may lead to this stress. Maternal factors, such as genotypic predisposition to endothelial disease and impairment of antioxidant defence leading to a limited capacity of the maternal clearing system, which cannot cope with the increasing charge of apoptotic cell debris, may be related to the development of late onset preeclampsia [20]. It remains unclear, however, what factors are critical to directing the cascade of events towards the spontaneous onset of labor. In the absence of spontaneous labor, further deterioration will eventually lead to the induction of labour.

Factors involved in the regulation of normal development of placental function, its possible aberrations, and their significance in the context of diverse abnormalities of pregnancy have been the focus of the recent “Global Pregnancy Collaboration symposium on placental health” [22].

## From the Vice-Editor

We are honored to have received a manuscript from Professor Henning Schneider, a distinguished author, renown Swiss obstetrician-gynecologist, and perhaps foremost an acclaimed placentologist. Therefore, it is not surprising that a sophisticated look at preeclampsia be presented from a placental perspective.

The current contribution gives the reader a glimpse of an outstanding lecture presented by Professor Schneider during an international obstetric-gynecologic conference held in Katowice, Poland in April 2017. The manuscript combines with ease the principal clinical aspects of preeclampsia with emerging cellular and molecular findings. We witness how the pathogenesis of this disorder is being better and better understood with the work highlighting some of the most important achievements in this area.

**Professor Maciej Jóźwik**
